# Supplementary material for: Evolutionary trade-offs in dormancy phenology
Source: eLife. 2024 Apr 26;12:RP89644. doi: 10.7554/eLife.89644 (PMC11052570; doi:10.7554/eLife.89644)
Supplement: Figure 3—source data 1. [file elife-89644-fig3-data1.docx]

Figure 3-source data 1 : Data on dependent and independent factors used in models 1. Protandry was used as the dependent factor and Body mass change during mating, late mating, body mass male, strategy fatstoring or foodstoring, minimum temperature, precipitation and dimorphism of body mass at emergence were considered as independent factors. Protandry was calculated as follows: Female Julian date – male Julian date. The exact hibernation phenology data for *Cricetus cricetus* have been confirmed by the authors. See materials and methods for the acquisition of minimum temperature data.

| species | Late mating | Food/  Fat | protandry | Body mass change during mating | Body mass male | Dimorphism body mass emergence | precipitation | Min temperature |
| --- | --- | --- | --- | --- | --- | --- | --- | --- |
| Callospermophilus lateralis | 1^1^ | Fat | 21^1^ | -11,126^2^ | 173^2^ | 1,26^2^ | 411 | -7 |
| Callospermophilus saturatus | 1^3^ | Food | 4^3^ | -6,38^3^ | 248^3^ | 1,45^3^ | 863 | -7,3 |
| Chaetodipus formosus | 4^4^ | Food | 14^4^ | 0,8^4^ | 18,6^4^ | 1,12^4^ | 863 | -7,3 |
| Cricetus cricetus | 2,5^5,6^ | Food | 20^7^ | -5,6^8^ | 400,9^8^ | 1,58^6,8^ | 595 | -2,7 |
| Cynomys leucurus | 1^9^ | Fat | 21^9,10^ | -21,61^9^ | 829,4^9^ | 1,69^9^ | 337 | -11,5 |
| Erinaceus europaeus | 1^11,12^ | Fat | 17,6^12,13^ | -12,5^12,13^ | 1028,67^12,13^ | 1,32^12,13^ | 803 | -5,3 |
| Glis glis | 1^14^ | Fat | 30^15^ | -8,6^14^ | 105,5^14^ | 1,1^14^ | 758 | -2,5 |
| Ictidomys parvidens | 6^16^ | Fat | 7,5^16^ | -10,5^16^ | 192^16^ | 1,27^16^ | 347 | -2,4 |
| Marmota monax | 1^17^ | Fat | 22^18^ | -6,2^17^ | 2701^17^ | 1,03^17^ | 1156 | -11,5 |
| Microcebus murinus | 1^19^ | Fat | 48^19^ | -20,7^20^ | 62^20^ | 1,02^20^ | 922 | 14,6 |
| Perognathus longimembris | 4^4^ | Food | 14^21^ | 3,2^21^ | 7^21^ | 1,06^21^ | 863 | -7,3 |
| Poliocitellus franklinii | 1^22,23^ | Fat | 11^22,23^ | -2,62^22,23^ | 417,1^22,23^ | 1,27^22,23^ | 517 | -21,1 |
| Spermophilus citellus | 1^24^ | Fat | 23^25^ | -9,8^25^ | 316,26^25^ | 1,68^25^ | 556 | -2,5 |
| Spermophilus xanthoprymnus | 1^24^ | Fat | 7,5^24^ | -11,62^24^ | 292^24^ | 1,69^24^ | 407 | -5,2 |
| Tachyglossus aculeatus | 1^26^ | Fat | 45^26^ | -12,7^27^ | 3950^27^ | 1,06^27^ | 625 | 3,6 |
| Tamias amoenus | 1^21^ | Food | 10,5^21^ | -5,4^21^ | 42,3^21^ | 0,91^21^ | 846 | -7,3 |
| Tamias sibiricus | 1^28^ | Food | 20^28^ | 1,5^29^ | 90,6^29^ | 0,96^29^ | 942 | -9,4 |
| Urocitellus armatus | 1^24^ | Fat | 3,66^30^ | 6,82^30^ | 333,7^30^ | 1,25^30^ | 402 | -13 |
| Urocitellus beldingi | 1^24^ | Fat | 9^31^ | -0,4^31^ | 240,1^31^ | 1,14^31^ | 568 | -12,4 |
| Urocitellus brunneus | 1^32^ | Fat | 9^32^ | -6,57^33^ | 175^33^ | 1,44^33^ | 610 | -11 |
| Urocitellus columbianus | 1^24^ | Fat | 7^34^ | -0,4^34^ | 430^34^ | 1,18^34^ | 346 | -17,5 |
| Urocitellus elegans | 1^35^ | Fat | 17,3^35^ | -3,8^35^ | 249,3^35^ | 1,37^35^ | 281 | -16,6 |
| Urocitellus mollis | 1^36^ | Fat | 15^36^ | 4,17^37^ | 181^37^ | 1,49^37^ | 248 | -5,4 |
| Urocitellus parryii | 1^24^ | Food | 14^38^ | -21,6^39^ | 941^39^ | 1,58^39^ | 237 | -30,2 |
| Urocitellus richardsonii | 1^24^ | Food | 16^40–43^ | -10^41,43^ | 392,5^41,43^ | 1,7^41,43^ | 371,5 | -14,4 |
| Xerospermophilus tereticaudus | 4^44^ | Fat | 5,3^44^ | 4,2^44^ | 142^44^ | 1,27^44^ | 250 | 2,4 |
| Zapus hudsonius | 1^45^ | Fat | 14^38^ | -2,67^45^ | 17^45^ | 1,02^45^ | 837 | -8,5 |
| Zapus princeps | 1^46^ | Fat | 10,33^46^ | -7,91^47^ | 22,48^47^ | 0,95^47^ | 570 | -14,1 |

References

1. Bronson, M. T. Altitudinal variation in emergence time of golden-mantled ground squirrels (Spermophilus lateralis). *Journal of Mammalogy* **61**, 124–126 (1980).

2. McKeever, S. The biology of the golden-mantled ground squirrel, Citellus lateralis. *Ecological Monographs* **34**, 383–401 (1964).

3. Kenagy, G. J., Sharbaugh, S. M. & Nagy, K. A. Annual cycle of energy and time expenditure in a golden-mantled ground squirrel population. *Oecologia* **78**, 269–282 (1989).

4. Kenagy, G. J. & Bartholomew, G. A. Seasonal Reproductive Patterns in Five Coexisting California Desert Rodent Species: Ecological Archives M055-002. *Ecological Monographs* **55**, 371–397 (1985).

5. Siutz, C., Valent, M., Ammann, V., Niebauer, A. & Millesi, E. Sex-specific effects of food supplementation on hibernation performance and reproductive timing in free-ranging common hamsters. *Sci Rep* **8**, 1–10 (2018).

6. Hufnagl, S., Franceschini-Zink, C. & Millesi, E. Seasonal constraints and reproductive performance in female Common hamsters (Cricetus cricetus). *Mammalian Biology* **76**, 124–128 (2011).

7. Siutz, C., Franceschini, C. & Millesi, E. Sex and age differences in hibernation patterns of common hamsters: adult females hibernate for shorter periods than males. *J Comp Physiol B* **186**, 801–811 (2016).

8. Lebl, K. & Millesi, E. Yearling male Common hamsters and the trade-off between growth and reproduction. *Biosystematics and Ecology Series* **25**, 115–126 (2008).

9. Bakko, E. B. & Brown, L. N. Breeding Biology of The White-Tailed Prairie Dog, Cynomys Leucurus, in Wyoming. *Journal of Mammalogy* **48**, 100–112 (1967).

10. Clark, T. W. Ecology and ethology of the white-tailed prairie dog (Cynomys leucurus). *Milwaukee Public Museum Publications in Biology and Geology* **3**, 1–96 (1977).

11. Rautio, A., Valtonen, A. & Kunnasranta, M. The effects of sex and season on home range in European hedgehogs at the northern edge of the species range. in *Annales Zoologici Fennici* vol. 50 107–123 (BioOne, 2013).

12. Haigh, A., O’Riordan, R. M. & Butler, F. Nesting behaviour and seasonal body mass changes in a rural Irish population of the Western hedgehog (Erinaceus europaeus). *Acta Theriologica* **57**, 321–331 (2012).

13. Rautio, A., Valtonen, A., Auttila, M. & Kunnasranta, M. Nesting patterns of European hedgehogs (Erinaceus europaeus) under northern conditions. *Acta theriologica* **59**, 173–181 (2014).

14. Bieber, C. Population dynamics, sexual activity, and reproduction failure in the fat dormouse (Myoxus glis). *Journal of Zoology* **244**, 223–229 (1998).

15. Bieber, C. & Ruf, T. Seasonal timing of reproduction and hibernation in the edible dormouse (Glis glis). *Life in the cold: Evolution, mechanism, adaptation, and application* 113–125 (2004).

16. Schwanz, L. E. Annual cycle of activity, reproduction, and body mass in Mexican ground squirrels (Spermophilus mexicanus). *Journal of Mammalogy* **87**, 1086–1095 (2006).

17. Maher, C. R. & Duron, M. Mating system and paternity in woodchucks (Marmota monax). *Journal of Mammalogy* **91**, 628–635 (2010).

18. Maher, C. R. Social organization in woodchucks (Marmota monax) and its relationship to growing season. *Ethology* **112**, 313–324 (2006).

19. Schmid, J. Sex-specific differences in activity patterns and fattening in the gray mouse lemur (Microcebus murinus) in Madagascar. *Journal of Mammalogy* **80**, 749–757 (1999).

20. Schmid, J. & Kappeler, P. M. Fluctuating sexual dimorphism and differential hibernation by sex in a primate, the gray mouse lemur (Microcebus murinus). *Behavioral Ecology and Sociobiology* **43**, 125–132 (1998).

21. Kenagy, G. J. & Barnes, B. M. Seasonal Reproductive Patterns in Four Coexisting Rodent Species from the Cascade Mountains, Washington. *J Mammal* **69**, 274–292 (1988).

22. Iverson, S. L. & Turner, B. N. Natural history of a Manitoba population of Franklin’s ground squirrels. *Canadian Field-Naturalist* **86**, 145–149 (1972).

23. Choromanski-Norris, J., Fritzell, E. K. & Sargeant, A. B. Seasonal Activity Cycle and Weight Changes of the Franklin’s Ground Squirrel. *American Midland Naturalist* **116**, 101 (1986).

24. Gür, H. & Gür, M. K. Annual cycle of activity, reproduction, and body mass of Anatolian ground squirrels (Spermophilus xanthoprymnus) in Turkey. *Journal of Mammalogy* **86**, 7–14 (2005).

25. Millesi, E., Strijkstra, A. M., Hoffmann, I. E., Dittami, J. P. & Daan, S. Sex and Age Differences in Mass, Morphology, and Annual Cycle in European Ground Squirrels, Spermophilus citellus. *J Mammal* **80**, 218–231 (1999).

26. Nicol, S. C., Morrow, G. E. & Harris, R. L. Energetics meets sexual conflict: The phenology of hibernation in Tasmanian echidnas. *Functional Ecology* **33**, 2150–2160 (2019).

27. Nicol, S. C., Andersen, N. A., Morrow, G. E. & Harris, R. L. Spurs, sexual dimorphism and reproductive maturity in Tasmanian echidnas (Tachyglossus aculeatus setosus). *Australian Mammalogy* **41**, 161–169 (2018).

28. Kawamichi, M. Nest Structure Dynamics and Seasonal Use of Nests by Siberian Chipmunks (Eutamias sibiricus). *J Mammal* **70**, 44–57 (1989).

29. Kawamichi, M. Ecological Factors Affecting Annual Variation in Commencement of Hibernation in Wild Chipmunks (Tamias sibiricus). *J Mammal* **77**, 731–744 (1996).

30. Knopf, F. L. & Balph, D. F. Annual Periodicity of Uinta Ground Squirrels. *The Southwestern Naturalist* **22**, 213–224 (1977).

31. Morton, M. L. & Sherman, P. W. Effects of a spring snowstorm on behavior, reproduction, and survival of Belding’s ground squirrels. *Canadian Journal of Zoology* **56**, 2578–2590 (1978).

32. Goldberg, A. R. *Diet, disease, and hibernation behavior of northern Idaho ground squirrels*. (University of Idaho, 2018).

33. Barrett, J. S. Population viability of the southern Idaho ground squirrel (Spermophilus brunneus endemicus): effects of an altered landscape. (2005).

34. Raveh, S. *et al.* Mating order and reproductive success in male Columbian ground squirrels (Urocitellus columbianus). *Behavioral Ecology* **21**, 537–547 (2010).

35. Fagerstone, K. A. The annual cycle of Wyoming ground squirrels in Colorado. *Journal of Mammalogy* **69**, 678–687 (1988).

36. Van Horne, B., Schooley, R. L., Olson, G. S. & Burnham, K. P. Patterns of density, reproduction, and survival in Townsend’s ground squirrels. *Snake River Birds of Prey Area, research and monitoring, annual report. Edited by K. Steenhof. US Department of the Interior, Bureau of Land Management, Boise, Idaho* 158 (1993).

37. Van Horne, B., Olson, G. S., Schooley, R. L., Corn, J. G. & Burnham, K. P. EFFECTS OF DROUGHT AND PROLONGED WINTER ON TOWNSEND’S GROUND SQUIRREL DEMOGRAPHY IN SHRUBSTEPPE HABITATS. *Ecological Monographs* **67**, 295–315 (1997).

38. Sheriff, M. J. *et al.* Phenological variation in annual timing of hibernation and breeding in nearby populations of Arctic ground squirrels. *Proceedings of the Royal Society B: Biological Sciences* **278**, 2369–2375 (2011).

39. Buck, C. L. & Barnes, B. M. Annual cycle of body composition and hibernation in free-living arctic ground squirrels. *Journal of Mammalogy* **80**, 430–442 (1999).

40. Michener, G. R. & Locklear, L. Over-winter weight loss by Richardson’s ground squirrels in relation to sexual differences in mating effort. *Journal of Mammalogy* **71**, 489–499 (1990).

41. Michener, G. R. Sexual differences in reproductive effort of Richardson’s ground squirrels. *Journal of Mammalogy* **79**, 1–19 (1998).

42. Michener, G. R. Sexual differences in over-winter torpor patterns of Richardson’s ground squirrels in natural hibernacula. *Oecologia* **89**, 397–406 (1992).

43. Michener, G. R. & Locklear, L. Differential costs of reproductive effort for male and female Richardson’s ground squirrels. *Ecology* **71**, 855–868 (1990).

44. Munroe, K. E. & Koprowski, J. L. Annual cycles in the desert: body mass, activity and reproduction in round-tailed ground squirrels (Xerospermophilus tereticaudus). *THE SOCIOECOLOGY, MATING SYSTEM AND BEHAVIOR OF ROUND-TAILED GROUND SQUIRRELS (XEROSPERMOPHILUS TERETICAUDUS)* 29 (2011).

45. Hoyle, J. & Boonstra, R. Life history traits of the meadow jumping mouse, Zapus hudsonius, in southern Ontario. (1986).

46. Brown, L. N. Seasonal activity patterns and breeding of the western jumping mouse (Zapus princeps) in Wyoming. *American Midland Naturalist* 460–470 (1967).

47. Cranford, J. A. Ecological strategies of a small hibernator, the western jumping mouse *Zapus princeps*. *Can. J. Zool.* **61**, 232–240 (1983).
