# Supplementary material for: Evolutionary trade-offs in dormancy phenology
Source: eLife. 2024 Apr 26;12:RP89644. doi: 10.7554/eLife.89644 (PMC11052570; doi:10.7554/eLife.89644)
Supplement: Figure 4—source data 1. [file elife-89644-fig4-data1.docx]

Figure 4-source data 1 : Data on dependent and independent factors used in models 2. Sex difference in immergence was used as the dependent factor whereas strategy fatstoring or foodstoring, body mass change through the end of mating, body mass immergence, dimorphism at immergence maternal effort, maternal effort duration, female specific reproductive effort, minimum temperature and precipitation were considered as independent factors. The exact hibernation phenology data for *Cricetus cricetus* have been confirmed by the authors. Weaning body mass for *Zapus princeps* and *Urocitellus mollis* was calculated from body mass data at 52-60 days and 29 days, respectively, and corrected at 48 and 34 days, corresponding to weaning dates. For maternal effort duration of *Urocitellus brunneus* and *Ictidomys parvidens*, we used respectively the averages for the clade *Marmotini* obtained from Hayssen (2008). The body mass at weaning for *Urocitellus brunneus* correpsonds to the body mass at weaning of *Urocitellus townsendii* (phylogenetically close species) corrected for the difference in body mass of the females from Hayssen (2008) as body mass accounts for 76–84% of the variation in weaning mass in the clade *Marmotini* (Hayssen, 2008)

Sex difference in immergence was calculated as follows: female Julian date – male Julian date.

| Species | Food/  Fat- | Sex diff immerg | Minimum temper | Body mass change end of mating | Precipitation | Body mass immerg | Maternal effort duration | Littersize | Weaning mass | Body mass femelle | Dimorphism immergence | Specific reprod effort |
| --- | --- | --- | --- | --- | --- | --- | --- | --- | --- | --- | --- | --- |
| Callospermophilus saturatus | Food | 3^2^ | -7,3 | -13,1^2^ | 863 | 273^2^ | 70^1,3^ | 2,7^2^ | 76^2^ | 185^2^ | 0,97^2^ | 110,7 |
| Cricetus cricetus | Food | 27^4^ | -2,7 | 3,2^8^ | 595 | 344^5,6^ | 43^3^ | 7,31^5^ | 105^5^ | 245^6^ | 1,44^5,6^ | 313,2 |
| Cynomys leucurus | Fat | 11^10^ | -11,5 | -17,7^7^ | 337 | 1288^7^ | 67^1^ | 5,64^7^ | 198,5^7^ | 491^7^ | 1,55^7^ | 227,6 |
| Erinaceus europaeus | Fat | 15,7^8,9^ | -5,2 | -10,1^8,9^ | 803,5 | 1152^8,9^ | 72^2^ | 5^10^ | 235^11^ | 606^8,9^ | 1,16^8,9^ | 193,8 |
| Glis glis | Fat | 14^12^ | -2,5 | -2,5^13^ | 758 | 122,26^13^ | 58^3^ | 4,9^14^ | 39,2^15^ | 99,32^13^ | 1,21^13^ | 193,3 |
| Ictidomys parvidens | Fat | 15^16^ | -2,4 | 24,4^16^ | 347 | 282,5^16^ | 68^1^ | 6,4^16^ | 43,8^16^ | 187^16^ | 1,24^16^ | 149,9 |
| Marmota monax | Fat | -2^17^ | -11,5 | -6,2^18^ | 1156 | 3798,7^18^ | 76,05^1^ | 3,5^19^ | 490^17^ | 2610^18^ | 1,08^18^ | 65,7 |
| Microcebus murinus | Fat | 0^20^ | 14,6 | -13,5^21^ | 922 | 74,5^21^ | 98^3^ | 2^22^ | 36,6^23^ | 60^21^ | 0,71^21^ | 121,6 |
| Poliocitellus franklinii | Fat | 15,75^24,25^ | -21,1 | -4,5^24,25^ | 517 | 577,88^24,25^ | 58^1^ | 6,75^25^ | 101,33^1^ | 322^24,25^ | 1,26^24,25^ | 211,7 |
| Spermophilus citellus | Fat | -21,25^26^ | -2,5 | -13,3^26^ | 556 | 352,47^26^ | 64,5^1^ | 3,9^26^ | 51,5^27^ | 188^26^ | 1,31^26^ | 106,8 |
| Tachyglossus aculeatus | Fat | 24^28^ | 3,5 | -6,6^29^ | 625 | 4180^29^ | 167,5^30^ | 1^31^ | 1650^31^ | 3806^29^ | 1,13^29^ | 43,3 |
| Urocitellus armatus | Fat | -8,6^32^ | -13 | 2,2^32^ | 402 | 521,83^32^ | 45^1^ | 6^33^ | 60,3^34^ | 262^32^ | 1,32^32^ | 137,7 |
| Urocitellus beldingi | Fat | 20^35^ | -12,3 | -4,5^35^ | 568 | 381,8^35^ | 51,53^1^ | 6,31^36^ | 69^37^ | 217^35^ | 1,21^35^ | 200,6 |
| Urocitellus brunneus | Fat | -2^38^ | -11 | -6,5^39^ | 610 | 269,5^39^ | 66,5^1^ | 6,2^40^ | 36,55^1^ | 121^39^ | 1,35^39^ | 186,7 |
| Urocitellus columbianus | Fat | 11,7^41^ | -16,1 | 8,4^41^ | 693 | 579^41^ | 53,2^1^ | 3,6^42^ | 112,6^43^ | 380^41^ | 1,32^41^ | 106,6 |
| Urocitellus elegans | Fat | -9^44^ | -16,6 | -3,8^44^ | 281 | 363,68^44^ | 55,58^1^ | 5,88^45^ | 80,5^46^ | 203^44^ | 1,25^44^ | 233,1 |
| Urocitellus mollis | Fat | -3,66^47^ | -5,4 | -3,4^48^ | 248 | 227,83^48^ | 58^1^ | 8,1^47^ | 32,8^47^ | 122,5^48^ | 1,38^48^ | 216,8 |
| Urocitellus parryii | Food | -28^49^ | -30,2 | -21,2^50^ | 237 | 1034,74^50^ | 53 | 5,8 | 234,5 | 615^50^ | 1,05^50^ | 221,1 |
| Urocitellus richardsonii | Food | 12,16^51–54^ | -14,42 | -4,4^52,54^ | 370 | 524,52^52,54^ | 51,79 | 7,1 | 83,75 | 229^52,54^ | 1,30^52,54^ | 259,6 |
| Zapus hudsonius | Fat | 14^49^ | -8,5 | -9,5^55^ | 837 | 22,78^55^ | 47 | 5,5 | 7,8 | 16,7^55^ | 0,92^55^ | 256,8 |
| Zapus princeps | Fat | 0^56^ | -14,1 | -7,9^57^ | 570 | 35,24^57^ | 48 | 5,4 | 12,9 | 23^57^ | 1^57^ | 296,9 |

References

1. Hayssen, V. Reproductive Effort in Squirrels: Ecological, Phylogenetic, Allometric, and Latitudinal Patterns. *J Mammal* **89**, 582–606 (2008).

2. Kenagy, G. J., Sharbaugh, S. M. & Nagy, K. A. Annual cycle of energy and time expenditure in a golden-mantled ground squirrel population. *Oecologia* **78**, 269–282 (1989).

3. Magalhães, J. P. D. & Costa, J. A database of vertebrate longevity records and their relation to other life-history traits. *Journal of Evolutionary Biology* **22**, 1770–1774 (2009).

4. Siutz, C., Franceschini, C. & Millesi, E. Sex and age differences in hibernation patterns of common hamsters: adult females hibernate for shorter periods than males. *J Comp Physiol B* **186**, 801–811 (2016).

5. Hufnagl, S., Franceschini-Zink, C. & Millesi, E. Seasonal constraints and reproductive performance in female Common hamsters (Cricetus cricetus). *Mammalian Biology* **76**, 124–128 (2011).

6. Lebl, K. & Millesi, E. Yearling male Common hamsters and the trade-off between growth and reproduction. *Biosystematics and Ecology Series* **25**, 115–126 (2008).

7. Bakko, E. B. & Brown, L. N. Breeding Biology of The White-Tailed Prairie Dog, Cynomys Leucurus, in Wyoming. *Journal of Mammalogy* **48**, 100–112 (1967).

8. Rautio, A., Valtonen, A. & Kunnasranta, M. The effects of sex and season on home range in European hedgehogs at the northern edge of the species range. in *Annales Zoologici Fennici* vol. 50 107–123 (BioOne, 2013).

9. Haigh, A., O’Riordan, R. M. & Butler, F. Nesting behaviour and seasonal body mass changes in a rural Irish population of the Western hedgehog (Erinaceus europaeus). *Acta Theriologica* **57**, 321–331 (2012).

10. Kristiansson, H. Young production of European hedgehog in Sweden and Britain. *Acta Theriologica* **26**, 504–507 (1981).

11. Haigh, A. The ecology of the European hedgehog (Erinaceus europaeus) in rural Ireland. (University College Cork, 2011).

12. Bieber, C. & Ruf, T. Seasonal timing of reproduction and hibernation in the edible dormouse (Glis glis). *Life in the cold: Evolution, mechanism, adaptation, and application* 113–125 (2004).

13. Bieber, C. Population dynamics, sexual activity, and reproduction failure in the fat dormouse (Myoxus glis). *Journal of Zoology* **244**, 223–229 (1998).

14. Vekhnik, V. A., Ruf, T. & Bieber, C. A Review on the Edible dormouse reproduction (Glis glis Linnaeus, 1766). *Journal of Wildlife and Biodiversity* **6**, 24–45 (2022).

15. Kryštufek, B. Glis glis (Rodentia: Gliridae). *Mammalian Species* **42**, 195–206 (2010).

16. Schwanz, L. E. Annual cycle of activity, reproduction, and body mass in Mexican ground squirrels (Spermophilus mexicanus). *Journal of Mammalogy* **87**, 1086–1095 (2006).

17. Maher, C. R. Social organization in woodchucks (Marmota monax) and its relationship to growing season. *Ethology* **112**, 313–324 (2006).

18. Maher, C. R. & Duron, M. Mating system and paternity in woodchucks (Marmota monax). *Journal of Mammalogy* **91**, 628–635 (2010).

19. Maher, C. R. Effects of relatedness on social interaction rates in a solitary marmot. *Animal Behaviour* **78**, 925–933 (2009).

20. Schmid, J. Sex-specific differences in activity patterns and fattening in the gray mouse lemur (Microcebus murinus) in Madagascar. *Journal of Mammalogy* **80**, 749–757 (1999).

21. Schmid, J. & Kappeler, P. M. Fluctuating sexual dimorphism and differential hibernation by sex in a primate, the gray mouse lemur (Microcebus murinus). *Behavioral Ecology and Sociobiology* **43**, 125–132 (1998).

22. Eberle, M. & Kappeler, P. M. Sex in the dark: determinants and consequences of mixed male mating tactics in Microcebus murinus, a small solitary nocturnal primate. *Behav Ecol Sociobiol* **57**, 77–90 (2004).

23. Zimmermann, E., Radespiel, U., Mestre-Francés, N. & Verdier, J.-M. 8 Life history variation in mouse lemurs (Microcebus murinus, M. lehilahytsara): the effect of environmental and phylogenetic determinants. *The Dwarf and Mouse Lemurs of Madagascar: Biology, Behavior and Conservation Biogeography of the Cheirogaleidae* **73**, 174 (2016).

24. Choromanski-Norris, J., Fritzell, E. K. & Sargeant, A. B. Seasonal Activity Cycle and Weight Changes of the Franklin’s Ground Squirrel. *American Midland Naturalist* **116**, 101 (1986).

25. Iverson, S. L. & Turner, B. N. Natural history of a Manitoba population of Franklin’s ground squirrels. *Canadian Field-Naturalist* **86**, 145–149 (1972).

26. Millesi, E., Strijkstra, A. M., Hoffmann, I. E., Dittami, J. P. & Daan, S. Sex and Age Differences in Mass, Morphology, and Annual Cycle in European Ground Squirrels, Spermophilus citellus. *J Mammal* **80**, 218–231 (1999).

27. Huber, S., Hoffmann, I. E., Millesi, E., Dittami, J. & Arnold, W. Explaining the seasonal decline in litter size in European ground squirrels. *Ecography* **24**, 205–211 (2001).

28. Nicol, S. C., Morrow, G. E. & Harris, R. L. Energetics meets sexual conflict: The phenology of hibernation in Tasmanian echidnas. *Functional Ecology* **33**, 2150–2160 (2019).

29. Nicol, S. C., Andersen, N. A., Morrow, G. E. & Harris, R. L. Spurs, sexual dimorphism and reproductive maturity in Tasmanian echidnas (Tachyglossus aculeatus setosus). *Australian Mammalogy* **41**, 161–169 (2018).

30. Morrow, G., Andersen, N. A. & Nicol, S. C. Reproductive strategies of the short-beaked echidna–a review with new data from a long-term study on the Tasmanian subspecies (Tachyglossus aculeatus setosus). *Australian Journal of Zoology* **57**, 275–282 (2009).

31. Nicol, S. & Andersen, N. A. The life history of an egg-laying mammal, the echidna (Tachyglossus aculeatus). *Ecoscience* **14**, 275 (2007).

32. Knopf, F. L. & Balph, D. F. Annual Periodicity of Uinta Ground Squirrels. *The Southwestern Naturalist* **22**, 213–224 (1977).

33. Slade, N. A. & Balph, D. F. Population Ecology of Uinta Ground Squirrels. *Ecology* **55**, 989–1003 (1974).

34. Eshelman, B. D. & Sonnemann, C. S. Spermophilus armatus. *Mamm Species* 1–6 (2000) doi:10.2307/0.637.1.

35. Morton, M. L. & Sherman, P. W. Effects of a spring snowstorm on behavior, reproduction, and survival of Belding’s ground squirrels. *Canadian Journal of Zoology* **56**, 2578–2590 (1978).

36. Morton, M. L. & Gallup, J. S. Reproductive cycle of the Belding ground squirrel (Spermophilus beldingi beldingi): seasonal and age differences. *The Great Basin Naturalist* 427–433 (1975).

37. Morton, M. L. & Tung, H. L. Growth and development in the Belding ground squirrel (Spermophilus beldingi beldingi). *Journal of Mammalogy* **52**, 611–616 (1971).

38. Goldberg, A. R. *Diet, disease, and hibernation behavior of northern Idaho ground squirrels*. (University of Idaho, 2018).

39. Barrett, J. S. Population viability of the southern Idaho ground squirrel (Spermophilus brunneus endemicus): effects of an altered landscape. (2005).

40. Yensen, E. & Sherman, P. W. Spermophilus brunneus. *Mamm Species* 1–5 (1997) doi:10.2307/3504405.

41. Raveh, S. *et al.* Mating order and reproductive success in male Columbian ground squirrels (Urocitellus columbianus). *Behavioral Ecology* **21**, 537–547 (2010).

42. Neuhaus, P. Parasite removal and its impact on litter size and body condition in Columbian ground squirrels (Spermophilus columbianus). *Proc. R. Soc. Lond. B* **270**, (2003).

43. Skibiel, A. L., Dobson, F. S. & Murie, J. O. Maternal influences on reproduction in two populations of Columbian ground squirrels. *Ecological Monographs* **79**, 325–341 (2009).

44. Fagerstone, K. A. The annual cycle of Wyoming ground squirrels in Colorado. *Journal of Mammalogy* **69**, 678–687 (1988).

45. Clark, T. W. Richardson’s ground squirrel (Spermophilus richardsonii) in the Laramie Basin, Wyoming. *The Great Basin Naturalist* 55–70 (1970).

46. Clark, T. W. Early growth, development, and behavior of the Richardson ground squirrel (Spermophilus richardsoni elegans). *American Midland Naturalist* 197–205 (1970).

47. Van Horne, B., Schooley, R. L., Olson, G. S. & Burnham, K. P. Patterns of density, reproduction, and survival in Townsend’s ground squirrels. *Snake River Birds of Prey Area, research and monitoring, annual report. Edited by K. Steenhof. US Department of the Interior, Bureau of Land Management, Boise, Idaho* 158 (1993).

48. Van Horne, B., Olson, G. S., Schooley, R. L., Corn, J. G. & Burnham, K. P. EFFECTS OF DROUGHT AND PROLONGED WINTER ON TOWNSEND’S GROUND SQUIRREL DEMOGRAPHY IN SHRUBSTEPPE HABITATS. *Ecological Monographs* **67**, 295–315 (1997).

49. Sheriff, M. J. *et al.* Phenological variation in annual timing of hibernation and breeding in nearby populations of Arctic ground squirrels. *Proceedings of the Royal Society B: Biological Sciences* **278**, 2369–2375 (2011).

50. Buck, C. L. & Barnes, B. M. Annual cycle of body composition and hibernation in free-living arctic ground squirrels. *Journal of Mammalogy* **80**, 430–442 (1999).

51. Michener, G. R. & Locklear, L. Over-winter weight loss by Richardson’s ground squirrels in relation to sexual differences in mating effort. *Journal of Mammalogy* **71**, 489–499 (1990).

52. Michener, G. R. Sexual differences in reproductive effort of Richardson’s ground squirrels. *Journal of Mammalogy* **79**, 1–19 (1998).

53. Michener, G. R. Sexual differences in over-winter torpor patterns of Richardson’s ground squirrels in natural hibernacula. *Oecologia* **89**, 397–406 (1992).

54. Michener, G. R. & Locklear, L. Differential costs of reproductive effort for male and female Richardson’s ground squirrels. *Ecology* **71**, 855–868 (1990).

55. Hoyle, J. & Boonstra, R. Life history traits of the meadow jumping mouse, Zapus hudsonius, in southern Ontario. (1986).

56. Brown, L. N. Seasonal activity patterns and breeding of the western jumping mouse (Zapus princeps) in Wyoming. *American Midland Naturalist* 460–470 (1967).

57. Cranford, J. A. Ecological strategies of a small hibernator, the western jumping mouse *Zapus princeps*. *Can. J. Zool.* **61**, 232–240 (1983).
